# Case report: Deep vein thrombosis as the sole clinical feature of Behcet’s syndrome

**DOI:** 10.3389/fmed.2023.1276867

**Published:** 2023-12-08

**Authors:** Abdelrahman Omara, Mohamed Alkhuboli, Javaid Nauman, Shamma Al Nokhatha, Mozah Almarshoodi

**Affiliations:** ^1^Department of Academic Affairs, Ambulatory Healthcare Services, Al Ain, United Arab Emirates; ^2^Department of Ambulatory Medicine - Medical Affairs, Tawam Hospital, Al Ain, United Arab Emirates; ^3^Institute of Public Health, College of Medicine and Health Sciences, United Arab Emirates University, Al Ain, United Arab Emirates; ^4^Faculty of Medicine and Health Sciences, Department of Circulation and Medical Imaging, Norwegian University of Science and Technology, Trondheim, Norway; ^5^Healthy Living for Pandemic Event Protection (HL-PIVOT) Network, Chicago, IL, United States; ^6^Rheumatology Department, Tawam Hospital, Al Ain, United Arab Emirates; ^7^College of Medicine and Health Sciences, UAE University, Al Ain, United Arab Emirates; ^8^Hematology Department, Tawam Hospital, Al Ain, United Arab Emirates

**Keywords:** Behcet’s syndrome, atypical presentation, systemic vasculitis, deep vein thrombosis, HLA-B51, pathergy test, breakthrough thrombosis

## Abstract

**Introduction:**

Behcet’s syndrome is a rare, chronic, systemic condition often categorized within the group of vasculitides. It presents a diagnostic challenge due to its varied clinical manifestations and the absence of a definitive laboratory test. Its etiology remains unclear but may involve genetic, infectious, and environmental factors.

**Case presentation:**

We report the case of a 16-year-old male who presented with deep vein thrombosis, followed by recurrent episodes of breakthrough thrombosis, despite adequate anticoagulant therapy. The patient did not meet the International Study Group (ISG) criteria nor the International Criteria for Behcet’s syndrome (ICBD) due to the absence of characteristic features such as oral aphthous ulcers, genital ulcers, and uveitis. Later in the disease course, after ruling out other causes of breakthrough thrombosis, he tested positive for HLA-B51, an allele associated with Behcet’s syndrome, and exhibited a pathergy reaction.

**Discussion:**

The patient’s clinical course underlines the diagnostic complexity associated with Behcet’s syndrome and highlights the importance of maintaining a broad differential diagnosis in cases of recurrent thrombosis. Although HLA-B51 testing is not routinely recommended, it played a pivotal role in our case, underscoring the value of an integrated diagnostic approach. Furthermore, this case reinforces the potential for atypical presentations of Behcet’s syndrome, necessitating vigilant clinical awareness. After establishing the diagnosis, we successfully treated the patient with immunosuppressive therapy, significantly improving his condition.

## Introduction

Behcet’s syndrome is an autoinflammatory condition, often categorized within the group of vasculitides, affecting multiple organ systems. Its onset often occurs in genetically predisposed individuals—such as those with the HLA-B51 allele—and is believed to be triggered by environmental factors and infections. The precise pathophysiology of Behcet’s syndrome remains elusive (1). Behcet’s syndrome commonly manifests during the third decade of life, although it can present at any age (2). Its prevalence varies across different regions worldwide, with reported rates of 119.8 cases per 100,000 inhabitants in Turkey, 31.8 in the Middle East, 4.5 in Asia, and 3.3 in Europe (3). Common features include recurrent oral aphthous ulcers, genital ulcers, eye inflammation, and skin lesions (2). Behcet’s syndrome is marked by its impact on multiple organ systems, with significant vascular manifestations that are major predictors of both mortality and morbidity. Diagnosis, in the absence of a pathognomonic test, leans heavily on clinical criteria. The two pivotal criteria are the International Study Group (ISG) and the International Criteria for Behcet’s Disease (ICBD) (4). This diagnostic complexity becomes even more pronounced when considering the vascular manifestations that occur in 12–15% of cases. Of these, deep vein thrombosis (DVT) is the predominant vascular manifestation, observed in 67.1% of patients. Notably, 74.6% encounter their first vascular event within 5 years of disease onset, and 10.8% experience it even before satisfying the full ISG criteria (5, 6). Considering the infrequency of vascular manifestations and their diverse presentations, deep vein thrombosis (DVT) as an isolated symptom can pose a diagnostic challenge, underscoring the uniqueness of our case.

The accurate and timely treatment of Behcet’s syndrome relies on recognizing both typical and atypical presentations, requiring clinical vigilance and a thorough understanding of its varied manifestations. Such awareness among clinicians can notably improve patient prognosis (4). In this case report, we highlight an unusual presentation of Behcet’s syndrome in a 16-year-old male who solely presented with recurrent deep vein thrombosis (DVT). This singular manifestation poses unique diagnostic challenges, offering an opportunity to explore the complexities of Behcet’s syndrome further. Our findings contribute to the growing understanding of atypical presentations, emphasizing the importance of a comprehensive approach to diagnosis and management.

## Case presentation

A 16-year-old male was referred to our hospital for investigation and management of left femoral deep vein thrombosis (DVT) that had developed 1 month prior. Initially treated with enoxaparin 50 mg twice daily, he was later switched to rivaroxaban 20 mg once daily. Upon his visit to our hematology clinic, he reported persistent pain in his left leg and a new onset of pain and swelling in his right leg, despite adherence to his rivaroxaban regimen. An emergent Doppler ultrasound was performed, confirming the diagnosis of bilateral breakthrough thrombosis in the femoral veins. Breakthrough thrombosis refers to the occurrence of thrombotic events, such as the formation of a blood clot, despite the patient receiving therapeutic anticoagulation. This finding necessitated the patient’s prompt admission to the hospital for further management.

Three months prior, the patient had flu-like symptoms and acute otitis media, following a SARS-CoV-2 booster shot. He had no other episodes of thrombosis in his personal history, apart from the recent event. He denied any history of connective tissue diseases, oral or genital ulcers, Raynaud’s phenomenon, joint pain, or photosensitivity. He also denied using any other medications or supplements. There were no lifestyle factors such as smoking or recent periods of immobility. Furthermore, there were no symptoms suggestive of pulmonary embolism or stroke. He also denied any unusual or excessive bleeding episodes. The family history was negative for thrombosis, cancer, autoimmune diseases, and known thrombophilia conditions.

During his admission, initial lab work showed elevated levels of D-dimer (1,430 mg/L), CRP (75.9 mg/L), ESR (58 mm/h), INR (1.58), PTT (41.7 s), and PT (16.3 s). The patient underwent extensive investigations to identify possible causes of his breakthrough thrombosis. For hereditary thrombophilia, tests were conducted for Factor V Leiden, factor II prothrombin genetic mutation, hyperhomocysteinemia, Protein C, Protein S, and Antithrombin III, all of which revealed normal levels. For potential acquired thrombophilia, tests were ordered for antiphospholipid syndrome (including lupus anticoagulant, anticardiolipin, and b2 macroglobulin) and vasculitis markers (C-ANCA and P-ANCA). The only positive result was a lupus anticoagulant test, which was considered as potentially a false positive due to the patient’s ongoing anticoagulant therapy.

The patient underwent a CT chest, which showed no evidence of pulmonary embolism. Additionally, a CT of the abdomen and pelvis with IV contrast was performed, revealing thrombosis in the bilateral external iliac veins and femoral veins. Following a notable improvement in his symptoms, the patient was discharged after 1 week on enoxaparin 50 mg twice daily and was scheduled for a follow-up appointment at the hospital’s hematology clinic.

**Figure 1:**
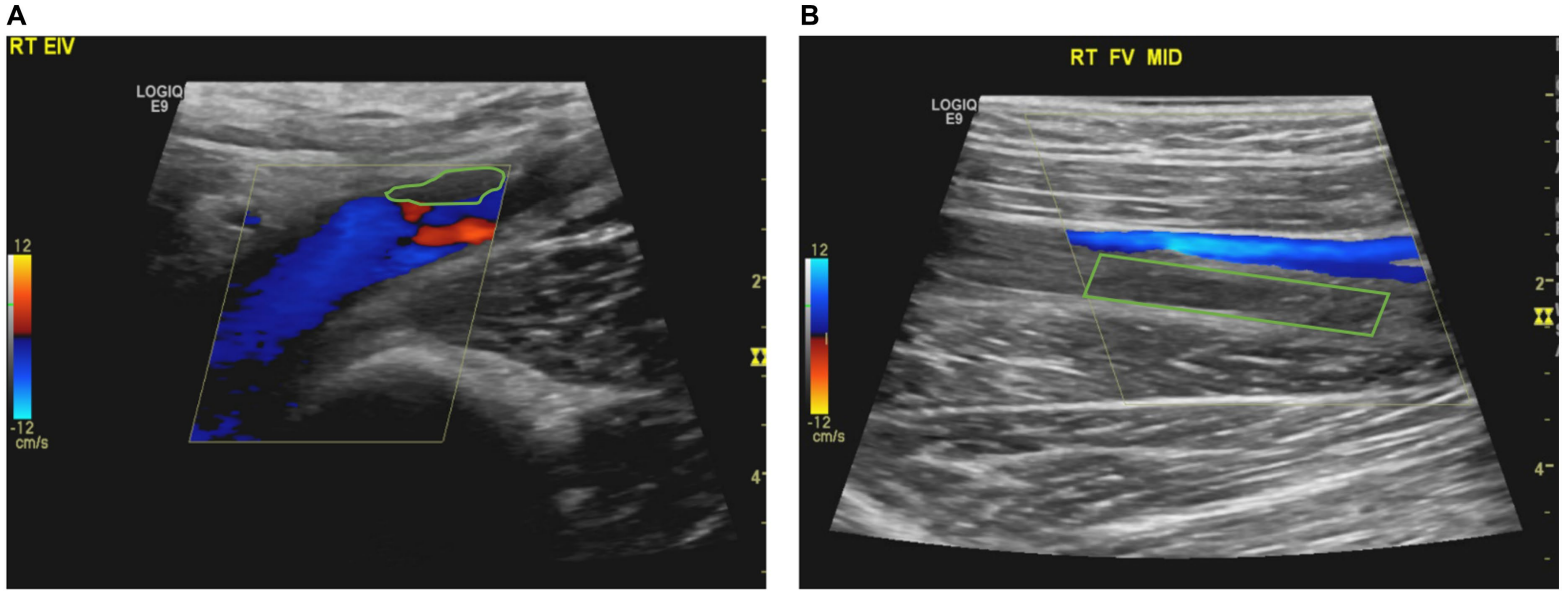
**(A)** Partial thrombosis/filling defect in the right external iliac vein. **(B)** Complete thrombosis in the mid-section of the right superficial femoral vein, with absence of flow.

Six weeks later, despite his initial treatment, the patient presented with worsening bilateral leg pain and swelling. Initial lab work showed elevated levels of D-dimer (2.86 mg/L), CRP (63.1 mg/L), ESR (75 mm/h), INR (1.23), PTT (37.7 s), PT (12.9 s). Lower extremities Doppler ultrasound was performed, showing bilateral iliac thrombosis with multiple new small venous thromboses in different territories (Figure 1). CT chest showed bilateral segmental/subsegmental pulmonary embolism involving the lower lobes. He was admitted for another episode of breakthrough thrombosis. A bilateral upper extremities Doppler ultrasound was performed to screen for widespread thrombosis and showed multiple thrombi across the distribution of upper extremities and neck veins. Enoxaparin dose was increased by 25% as per breakthrough thrombosis treatment guidelines with monitoring of anti-Xa. Additional potential causes of breakthrough thrombosis, such as paroxysmal nocturnal hemoglobinuria (PNH) and hematological malignancies, were ruled out with a peripheral blood flow cytometry analysis. Given the recurrent episodes of deep vein thrombosis and the exclusion of other causes of breakthrough thrombosis, Behcet’s syndrome was suspected. An HLA-B51 test was conducted, revealing a positive result, indicating the presence of the HLA-B51 allele, which supported this suspicion. During the examination, erythematous papulopustular lesions were incidentally observed on both arms. These lesions, which had developed in response to needle sticks from routine blood tests, were indicative of a pathergy reaction—a phenomenon associated with Behcet’s syndrome. With these findings, a diagnosis of atypical Bechet syndrome was made. Based on his diagnosis, prednisolone 50 mg (i.e., 1 mg/kg) and azathioprine 50 mg (i.e., 1 mg/kg) were initiated. Later, His D-dimer levels demonstrated a downward trend.

At the four-week follow-up post-diagnosis, the patient reported improvement in bilateral leg pain and swelling. A Doppler ultrasound of the lower extremities indicated improvement in the external iliac thrombosis, although not fully resolved. The Doppler ultrasound for the upper extremities showed the right brachial thrombosis remained unchanged. The azathioprine dosage was titrated up to 100 mg (i.e., 2 mg/kg) with an aim to reach 125 mg (i.e., 2.5 mg/kg). A structured tapering strategy was initiated for prednisolone, reducing the dose by 5 mg every 2 weeks.

At the second follow-up, 12 weeks post-diagnosis, the patient reported exacerbated pain and swelling in the left lower limb, especially while walking. Doppler ultrasound confirmed bilateral femoral thrombosis. Due to relapse of symptoms, prednisolone was adjusted from 30 mg to 40 mg (i.e., 0.75 mg/kg) and azathioprine was increased to 125 mg (i.e., 2.5 mg/kg).

At the third follow-up, fourteenth weeks post diagnosis, he exhibited side effects from azathioprine, manifesting as persistent diarrhea, nausea, and a mild elevation in liver enzymes. Consequently, the azathioprine dose was adjusted down to 100 mg (i.e., 2 mg/kg).

At the fourth follow-up, 16 weeks post diagnosis, he remained asymptomatic with both upper and lower extremity Doppler ultrasounds showing good recanalization. At this stage, prednisolone was tapered down from 40 mg (i.e., 0.75 mg/kg) by 5 mg every 2 weeks.

**Figure 2:**
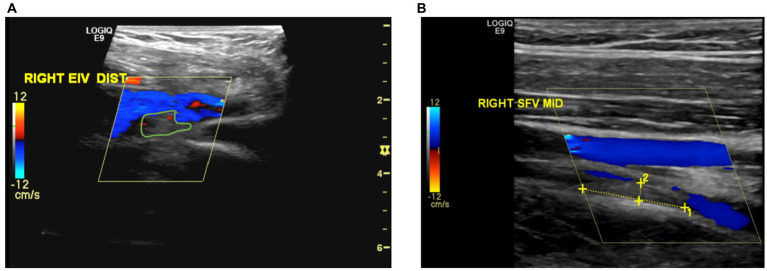
**(A)** Partial thrombosis or filling defect observed in the distal section of the right external iliac vein. **(B)** Recanalization observed in the previously completely thrombosed mid-section of the right superficial femoral vein.

**Figure 3:**
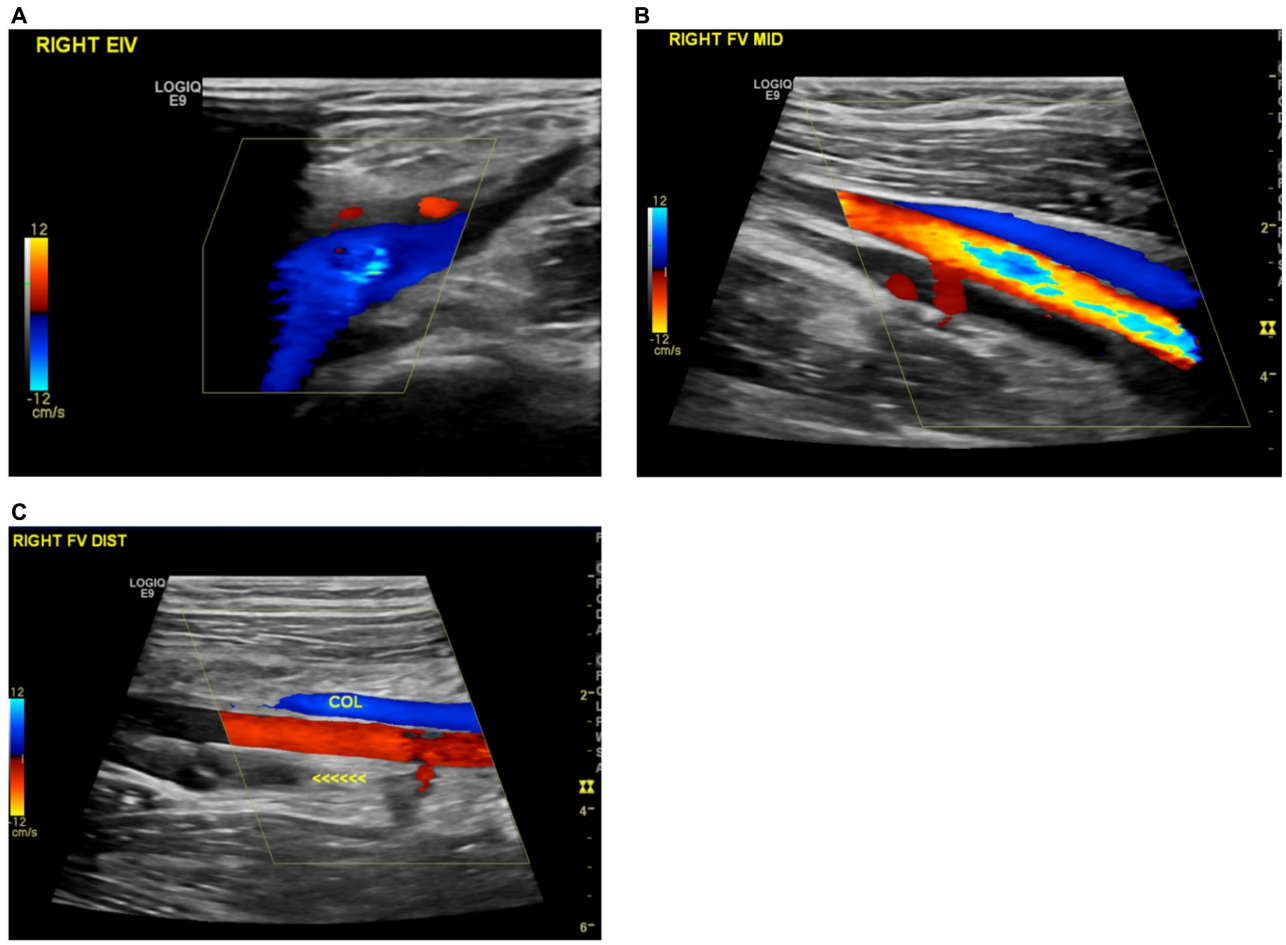
**(A)** Recanalization of the previously thrombosed right external iliac vein. **(B)** Improved recanalization observed in the mid-section of the right superficial femoral vein. **(C)** Formation of a new collateral vein in the right distal femoral vein.

During the fifth follow-up at 24 weeks post-diagnosis, the patient experienced a relapse, evidenced by progressive swelling in the bilateral lower limbs and the right upper limb. Doppler ultrasounds confirmed these findings (Figure 2). In response, the prednisolone dose was increased from 20 mg to 50 mg (i.e., 1 mg/kg) with continuation of azathioprine 100 mg (i.e., 2 mg/kg). Given the recurrent relapses of the disease, infliximab 5 mg/kg injections were initiated on a schedule of 0, 2, 4 weeks, followed by every 6 weeks thereafter. As of the current time, the patient has received a total of eight infliximab injections and is being maintained on azathioprine 100 mg daily. This regimen has resulted in marked clinical improvement, with an absence of active symptoms. Furthermore, ultrasound imaging of both the upper and lower extremities has revealed significant vascular recanalization with multiple collaterals (Figure 3).

## Discussion

We present the case of a 16-year-old male who sought medical help for left femoral vein thrombosis. Despite appropriate anticoagulant therapy, he experienced recurrent episodes of breakthrough thrombosis, leading us to question the underlying cause. After ruling out various common causes, we arrived at an atypical diagnosis of Behcet’s syndrome. This complex diagnostic approach underscores the importance of considering Behcet’s syndrome in perplexing cases of breakthrough thrombosis and highlights the challenges of diagnosing atypical presentations.

The patient’s past medical history of acute otitis media, while not explicitly linked to Behcet’s syndrome, falls within a broader spectrum of infections hypothesized to potentially trigger it. The role of infections, including Streptococcus species, in the pathogenesis of Behcet’s syndrome is still unclear. However, prevailing theories suggest a potential mechanism involving non-specific lymphocytic cell hypersensitivity or molecular mimicry (7). Alternatively, the potential of the SARS-CoV-2 booster shot to act as a trigger should not be overlooked, particularly in light of reported symptoms exacerbation in BD patients following vaccination (8).

Given the patient’s recurrent deep vein thrombosis (DVT), and after ensuring compliance with the appropriate anticoagulation dose, we initiated an extensive diagnostic workup for breakthrough thrombosis during the initial admission. The focus was on conditions such as hereditary and acquired thrombophilia, as these disorders can manifest solely with deep vein thrombosis and are sometimes found in patients with breakthrough events (9).

At that time, Behcet’s syndrome was not considered a probable cause of the patient’s breakthrough thrombosis. While the presence of vascular manifestations like thrombosis contributed to an ICBD score of 1, this score did not meet the threshold for a probable diagnosis of Behcet’s syndrome. Additionally, the absence of recurrent oral aphthous ulcers, a mandatory criterion in the ISG criteria, further ruled out Behcet’s syndrome during the first admission. This unique combination of factors added complexity to the diagnostic process, illustrating the challenges of diagnosing atypical presentations and emphasizing the need for a comprehensive approach to breakthrough thrombosis.

During the patient’s second admission, the workup extended to other causes of breakthrough thrombosis, such as paroxysmal nocturnal hemoglobinuria (PNH) and hematological malignancies. After excluding all these causes, including previously screened hereditary thrombophilia such as deficiencies in Protein C, Protein S, and Antithrombin III, Behcet’s syndrome was considered as a potential diagnosis. This case emphasizes the importance of maintaining a broad differential diagnosis, especially in challenging cases of recurrent thrombosis (9).

After the incidental finding of pathergy reaction, the patient’s ICBD score increased to 2. However, the patient still did not meet the criteria for a probable diagnosis according to the scoring interpretation, and the ISG criteria for Behcet’s syndrome were also not met. It is noteworthy that the pathergy test, while highly specific (> 90%) for Behcet’s syndrome, exhibits low sensitivity due to variations in prevalence across different geographical regions (e.g., 20% in the US vs. 75% in Turkey) (10, 11). Nevertheless, The high specificity of the pathergy test, coupled with a positive HLA-B51 result, may offer additional diagnostic value, though HLA-B51 is not routinely recommended for this purpose. The role of HLA-B51 is complex; while it is the predominant genetic determinant linked to Behcet’s Disease, it does not reliably predict specific clinical manifestations. Moreover, investigations into its precise role have yielded inconclusive results (12). Its presence in healthy individuals, as well as its detection in 50–80% of Behcet’s patients in endemic areas, further complicates its diagnostic utility (13, 14). These factors underscore the need for an integrated diagnostic approach that combines both clinical and laboratory findings.

Our case bears similarities with other instances where Behcet’s syndrome has manifested atypically, notably with vascular involvement as an initial presentation. For example, an 8-year-old patient initially presented with spontaneous bilateral lower extremity deep venous thromboses. However, unlike our case, this patient’s history was notable for recurrent oral aphthous ulcers, occurring 1 to 2 times a month for the past year, and erythema nodosum was found during the examination, leading to a diagnosis of Behcet’s syndrome (15). Similarly, a 22-year-old male presented with acute chest pain, dyspnea, and hemoptysis, symptoms that were eventually linked to Behcet’s syndrome with pulmonary artery thrombosis. His case was complicated by a long history of recurrent oral and genital ulcers, which had not led him to seek medical advice until the onset of severe symptoms (16).

Following the final diagnosis of Behcet’s syndrome, we adapted our treatment strategy to include immunosuppressive therapy with corticosteroids. This approach is crucial for mitigating recurrent episodes and post-thrombotic syndrome risks. In Behcet’s syndrome, deep vein thrombosis is usually due to inflammation of the vessel wall, not to a hypercoagulable state. However, the utility of anticoagulants in managing deep vein thrombosis associated with Behcet’s syndrome is still debated. If anticoagulants are to be used, it is vital to assess individual bleeding risks and rule out the presence of aortic aneurysms. There is no clear consensus favoring one immunomodulatory agent over another, but azathioprine, cyclosporine, and cyclophosphamide have been cited as viable options. Following recurrent episodes, we introduced infliximab—a TNF-alpha inhibitor—given its proven efficacy in treating resistant cases of deep vein thrombosis. The patient showed a significant response to infliximab, which led to the cessation of further thrombotic events and reinforced our Behcet’s syndrome diagnosis (17).

Upon reflection, we acknowledge that considering Behcet’s syndrome earlier, even without classic symptoms, could have expedited the diagnostic process and led to quicker symptom resolution, particularly in cases of recurrent unprovoked thrombosis. This highlights the potential limitations of relying solely on established diagnostic criteria and emphasizes the crucial role of clinical judgment, especially in atypical presentations. Therefore, increased clinical awareness about Behcet’s syndrome is warranted, particularly in complex or unusual presentations where common causes of thrombosis have been ruled out. This can aid in the timely and accurate diagnosis of Behcet’s syndrome.

## Data availability statement

The original contributions presented in the study are included in the article/supplementary material, further inquiries can be directed to the corresponding author.

## Ethics statement

The studies involving humans were approved by Tawam Human Research Ethics Committee (T-HREC). The studies were conducted in accordance with the local legislation and institutional requirements. Written informed consent for participation in this study was provided by the participants’ legal guardians/next of kin. Written informed consent was obtained from the individual(s), and minor(s)’ legal guardian/next of kin, for the publication of any potentially identifiable images or data included in this article.

## Author contributions

AO: Writing – original draft, Writing – review & editing. MohA: Writing – original draft, Writing – review & editing. JN: Writing – review & editing. SA: Writing – review & editing. MozA: Writing – review & editing.
